# Dibutyryl-cAMP affecting fat deposition of finishing pigs by decreasing the inflammatory system related to insulin sensitive or lipolysis

**DOI:** 10.1186/s12263-016-0531-5

**Published:** 2016-06-03

**Authors:** Xianyong Ma, Wei Fang, Zongyong Jiang, Li Wang, Xuefen Yang, Kaiguo Gao

**Affiliations:** 1 Institute of Animal Science, Guangdong Academy of Agricultural Sciences, 510640 Guangzhou, China; 2 The Key Laboratory of Animal Nutrition and Feed Science (South China) of Ministry of Agriculture, 510640 Guangzhou, China; 3 State Key Laboratory of Livestock and Poultry Breeding, 510640 Guangzhou, China; 4 Guangdong Public Laboratory of Animal Breeding and Nutrition, 510640 Guangzhou, China; 5 Guangdong Key Laboratory of Animal Breeding and Nutrition, 510640 Guangzhou, China

**Keywords:** Backfat thickness, Adipocyte cell, Gene microarray, Inflammatory system, Endocrine system

## Abstract

**Background:**

The mechanism of db-cAMP regulating fat deposition and improving lean percentage is unclear and needs to be further studied.

**Methods:**

Eighteen 100-day-old Duroc × Landrance × Large White barrows (49.75 ± 0.75 kg) were used for experiment 1, and 15 eighteen 135-day-old barrows (78.34 ± 1.22 kg) were used for experiment 2 to investigate the effects of dietary dibutyryl-cAMP (db-cAMP) on fat deposition in finishing pigs. Pigs were fed with a corn-soybean meal-based diet supplemented with 0 or 15 mg/kg db-cAMP, and both experiments lasted 35 days, respectively.

**Results:**

The results showed that db-cAMP decreased the backfat thickness, backfat percentage, and diameter of backfat cells without changing the growth performance or carcass characteristics in both experiments, and this effect was more marked in experiment 1 than in experiment 2; db-cAMP enhanced the activity of the growth hormone–insulin-like growth factor-1 (GH-IGF-1) axis and pro-opiomelanocortin (POMC) system in both experiments, which suppressed the accumulation of backfat deposition; microarray analysis showed that db-cAMP suppressed the inflammatory system within the adipose tissue related to insulin sensitivity, which also reduced fat synthesis.

**Conclusions:**

In summary, the effect of db-cAMP on suppressing fat synthesis and accumulation is better in the earlier phase than in the later phase of finishing pigs, and db-cAMP plays this function by increasing the activity of the GH-IGF-1 axis and POMC system, while decreasing the inflammatory system within the adipose tissue related to insulin sensitive or lipolysis.

## Background

Dibutyryl cAMP (db-cAMP), an orally active cell-permeant derivative of cAMP, has the same functions as endogenous cAMP, the latter playing a crucial role of signal transduction in numerous biological activities, such as regulating cell growth, enzyme activities, lipolysis, and gene expression [31, 38]. In recent years, dietary db-cAMP has been used in the pig production with beneficial effect on increasing the content of lean meat and decreasing fat in pig carcasses [39]. Our previous work [38] also showed that db-cAMP increased the lean percentage and decreased the backfat thickness, while it did not affect the growth performance or meat quality, indicating that db-cAMP may have potential for producing more lean meat as a feed additive in pig production. Some reports indicated that db-cAMP decreased the size of adipocytes, inhibited fat deposition in adipocytes, and reduced proliferation and differentiation of the preadipocytes [21, 38]. As cAMP is well-known for many hormones mediating signal transduction, and promoting lipolysis through adenylyl cyclase-cAMP-protein kinase pathway [4, 6], it seemed likely that exogenous db-cAMP also played functions through the endocrine and cell signaling pathway. Exogenous supplementation with cAMP or db-cAMP to anterior pituitary cells promoted the secretion of growth hormone [30] which is known to stimulate lean-tissue growth and inhibit adipose tissue growth [5]. Db-cAMP also increased the expression of beta-adrenoceptors and activated endogenous adenylate cyclase [7]. In addition, cAMP inhibited expression of genes such as *fatty acid synthase* (*FAS*) and *malate dehydrogenase* (*MDH*) in fat cells in vitro [11]. However, the mechanism of db-cAMP regulating fat deposition and improving lean percentage needs to be further studied. The present research has investigated the effect of dietary db-cAMP on concentrations of relevant hormones, metabolic indices, and gene expression profiles in backfat adipocytes of pigs to better understand the functional mechanisms of its action.

## Methods

### Pigs and diets

Eighteen 100-day-old Duroc × Landrance × Large White barrows (49.75 ± 0.75 kg) were used for experiment 1, and eighteen 135-day-old barrows (78.34 ± 1.22 kg) were used for experiment 2, each experiment lasting for 35 days and treated at the same condition. Other than for balancing on the basis of weight and ancestry, pigs were randomly assigned to 2 groups, controls, or supplemented with db-cAMP. Each treatment consisted of three replicate pens, each with three pigs. All pigs were housed in the animal facilities of the Institute of Animal Science in the Guangdong Academy of Agricultural Sciences. Pigs were fed with a corn-soybean meal-based diet (Table 1, meeting requirements for finishing pigs according to NRC (1998), without (as controls) or with 15 mg/kg db-cAMP (98 % purity, Hangzhou Meiya Biotechnology Co, Ltd, Hangzhou, China) as described by Wang et al. [38] (note: experiment 1 stands for the earlier phase of fattening pigs; experiment 2 stands for the later phase of fattening pigs).

### Feeding and slaughter procedure

Pigs were weighed at the end of the experiment, and feed consumed was recorded daily for each replicate to determine average daily gain (ADG), average daily feed intake (ADFI), and feed to gain (F:G). At the end of each experiment, pigs were fasted for 14 h, blood sampled, then immediately electro-stunned, and exsanguinated. All aspects of the experiment including transport and slaughtering procedures were carried out in accordance with the Chinese guidelines [29] and approved by Animal Ethical Committee of Institute of Animal Science, Guangdong Academy of Agricultural Sciences. Carcasses were weighed and split in the median plane. The dressing percentage, lean percentage, and fat percentage were measured using one side of the individual carcass. Backfat thickness was measured on the midline over the first, tenth, and last rib and cross-sectional area. The longissimus muscle area was measured at the junction of thoracic and lumbar vertebrae, by tracing onto sulfate paper followed by planimetry.

**Table 1:** Composition and nutrient levels of the basal diets for two stages of finishing pigs

Item	Experiment 1	Experiment 2	Chemical composition	Experiment 1	Experiment 2
Corn	67.700	69.300	Digestible energy (MJ/kg)	13.39	13.27
Soybean	25.900	20.700	Crude protein (%)	17.04	15.57
Wheat bran	3.645	7.645	Calcium (%)	0.66	0.55
Salt	0.370	0.370	Available energy (%)	0.24	0.19
Dicalcium Phosphate	0.900	0.600	Lysine (%)	0.97	0.85
Limestone	1.000	0.900	Methionine (%)	0.25	0.23
Lysine.HCl	0.130	0.130	Methionine + cysteine (%)	0.52	0.48
Mineral mix^*a*^	0.100	0.100			
Vitamin mix^*b*^	0.040	0.040			
Chloride choline (50 %)	0.100	0.100			
Antimildew agent	0.100	0.100			
Antioxidant agent	0.015	0.015			
Total	100.00	100.00			

^a^supplied 8 mg Cu (CuSO_4_.5H_2_O); 60 mg Fe (FeSO_4_.7H_2_O); 60 mg Zn (ZnSO_4_. H_2_O); 35 mg Mn (MnO); 0.35 mg I (KI ); 0.3 mg Se (NaSeO_3_) per kg of diet.

^b^supplied 5000 IU vitamin A; 700 IU vitamin D_3_; 25 mg vitamin E; 2.5 mg vitamin K_3_; 1.5 mg vitamin B_1_; 5 mg vitamin B_2_; 0.02 mg vitamin B_12_; 7.5 mg calcium pantothenate; 20 mg niacin; 0.5 mg folic acid; 0.04 mg biotin per kg of diet

### Sample collection

Blood was collected from the anterior vena cava using vacuum tubes (no anticoagulant), allowed to clot at room temperature for 120 min, and centrifuged for 5 min at 3000 × *g* at 4 °C, and serum was stored at −20 °C. Samples of longissimus muscle over the ninth to tenth ribs were immediately obtained and frozen in liquid N_2_ for measurement of intramuscular fat (IMF) content, enzyme activities, and messenger RNA (mRNA) analysis, and additional longissimus samples were held at 4 °C for meat quality measurements. Fresh samples of backfat adipose tissue (1 cm^3^) were fixed in 4 % paraformaldehyde in PBS (pH 7.3) for histology. Liver, pituitary, and hypothalamus tissue samples also were collected immediately and held, as described above, for mRNA extraction.

### Measurement of hormones and biochemical variables in plasma

The plasma concentrations of high-density lipoprotein (HDL), low-density lipoprotein (LDL), free fatty acid (FFA), cholesterol, and triglyceride (TG) were determined using an automatic analyzer (cx5, Beckman Coulter INC, Brea, CA), and the activity of lipase was determined using an ELISA kit (Luyu Bioengineering, Shanghai, China). The concentrations of cAMP, GH, IGF-I, IGFBP3, T3, T4, leptin, AD, and insulin were measured using ELISA kits (GBD Co, Ltd, USA).

### Meat quality measurements

The pH of muscle samples was measured at 45 min, 24 h, and 48 h postmortem using a pH meter (HI 8242C, Beijing Hanna Instruments Science & Technology, Beijing, China). Drip loss was measured, as described by Ma et al. [17]. Meat color CIE LAB values (L*, relative lightness; a*, relative redness; b*, relative yellowness) were determined on the transverse surface of the meat sample after it was cut and exposed to air for 45 min with a colorimeter (CR-410, Minolta, Suita-shi, Osaka, Japan); Shear force was measured using an Instron Universal Mechanical Machine (Instron model 4411; Instron, Canton, MA), as described by Ma et al. [17].

### Measurement of intramuscular fat content

The muscle samples were lyophilized and grounded to powders. The IMF content was measured by petroleum ether (30 to 60 °C boiling point) extraction using the Soxtec 2055 fat extraction system (Foss Tecator AB, Sweden), as described by Ma et al. [17].

### Diameter and the density of adipocytes

Fixed tissues were embedded in paraffin, sectioned at 5 micrometer (mm), dewaxed, and then stained with hematoxylin and eosin (Beijing Biosynthesis Biotechnology Co, Ltd, Beijing, China). The sections (ten sections per sample) were viewed at ×10 magnification using a Motic BA400 microscope, and the diameter and density of the adipocytes were determined with Motic image software (Motic-Optic Industrial Group Co. Ltd, Xiamen, China).

### Gene microarray analysis

Total RNA was isolated from backfat tissue from experiment 1 using TRIzol reagent (Invitrogen, Carlsbad, CA) according to the manufacturer’s instructions. The quality and quantity of RNA were assessed by OD_260_/OD_280_. Five micrograms of total RNA was converted to double-stranded complementary DNA (cDNA) using the RT-kit (QIAGEN, Shanghai, China) with an oligo (dT) primer containing a T7 RNA polymerase promoter. Biotin-labeled complementary RNA (cRNA) was synthesized from purified double-stranded cDNA using a Bio-Array high-yield RNA transcript labeling kit (QIAGEN). Approximately 20 mg cRNA was fragmented to 50–300 bases and hybridized to Porcine Oligo Microarray chips (Agilent, Santa Clara, CA). A total of six chips were used here: three replicates for controls and pigs receiving db-cAMP (mRNA was pooled for the three pigs each replicate). The hybridized arrays were washed, stained, and scanned following the Porcine Oligo Microarray GeneChip Expression Analysis manual.

### Real-time quantitative PCR of selected genes in backfat, liver, pituitary, and hypothalamus tissue

Total RNA was isolated (as above) from hypothalamus, pituitary, liver, and backfat tissue and stored at −80 °C. cDNA was produced using a commercial kit containing Reverse Transcriptase XL (AMV) and RNAsin (Invitrogen). Real-time PCR was performed using an ABI 7500 Mastercycler (Applied Biosystems, Foster City, CA) with qPCR Mix (TaKaRa, BIOINC, Japan). The gene (*ADCYAP1*, *GHRH*, *CRH*, *POMC*, *PC1*, *PC2*) expression levels were determined in hypothalamus tissue; the gene (*GHRHR*, *GH*, *CRHR*, *POMC*, *TRHR*, *TSH*) expression levels were determined in pituitary tissue; the gene (GHR, IGF-1) expression levels were determined in liver tissue. The selected genes (*SAL1*, *STAR*, *CYP2A19*, *STAT1*, *SYK*, *CCL2*, *AIF1*, *ITGB2*, *CCR1* and *CXCL2*) of backfat were chosen from the results of the chip analysis results and *beta-actin* (reference transcript gene) was designed from the GenBank sequences.

### Statistical analysis

Values were expressed as means ± SEM. Data were analyzed by *t* tests (Statistical Analysis Software version 8.1; SAS Institute, Inc, Cary, NC). Base on this, the effects of db-cAMP in experiments 1 and 2 were reanalyzed using two-way ANOVA followed by an appropriate post hoc *t* test. *P* values <0.05 were considered to be significant, and *P* values <0.01 were considered to be extremely significant.

Gene chip expression data analysis was performed using PCA project in the SAS system online. False discovery rate (FDR) correction was applied using the step-up method. Probe sets that met a FDR value of ≤0.05 and average fold change (FC) of at least 2 in either direction were selected for further study. The GenMAPP-CS software package (http://www.genmapp.org) was used for gene ontology (GO) and pathway analysis.

## Results

### Effect of dietary db-cAMP on growth performance and carcass characteristics

Dietary supplementation with 15 mg/kg db-cAMP did not affect (*P* > 0.05) final body weight, ADG, ADFI, or F:G ratio of the pigs in either experiments (Table 2). There were no significant effects of treatment on dressing percentage, longissimus muscle area, or lean percentage of the pigs in either experiment (Table 2). Dietary db-cAMP decreased the fat percentage of the pigs by 16.1 % in experiment 1 (135-day) and 12.1 % in experiment 2 (170-day) (*P* < 0.05), it also deceased the average backfat thickness by 22.4 % in experiment 1 and by 17.8 % in experiment 2 (*P* < 0.05), and the differences were greater in experiment 1 than that in experiment 2 (*P* < 0.05).

**Table 2:** Effects of db-cAMP on growth performance, carcass traits and meat quality in finishing pigs

Variable		Experiment 1		Experiment 2	
		Controls	db cAMP	Controls	db cAMP
Final weight (kg)		72.24±2.46	73.64±1.77	105.93±2.06	105.11±1.72
Average daily feed intake (kg)		2.32±0.15	2.25±0.17	2.85±0.08	2.72±0.10
Average daily gain (kg)		0.73±0.05	0.74±0.04	0.79±0.03	0.76±0.04
F/G		3.35±0.03	3.04±0.14	3.69±0.16	3.60±0.12
Dressing percentage (%)		73.80±1.25	73.40±0.56	78.22±0.87	77.84±0.76
Longissimus muscle area (cm^2^)		34.10±2.82	35.40±1.12	55.45±3.59	61.35±4.64
Lean percentage (%)		62.02±1.51	64.73±0.58	61.75±0.75	63.80±0.85
Fat percentage (%)#		19.55±1.06	16.41±0.32*	23.34±1.14	20.52±1.19*
Backfat thickness (cm)					
First rib#		3.70±0.19	2.89±0.32*	3.45±0.26	2.84±0.33
Tenth rib		1.67±0.11	1.33±0.16	1.89±0.21	1.56±0.33
Last rib		1.59±0.15	1.20±0.12	1.61±0.16	1.30±0.16
Average backfat thickness#		2.32±0.15	1.80±0.10**	2.31±0.09	1.90±0.08**
Drop loss (%) (24 h)		2.42±0.10	2.60±0.09	2.45±0.19	2.37±0.13
Intramuscular fat (%)		2.01±0.34	2.12±0.49	2.04±0.55	2.36±0.97
Shear force (48 h)		38.16±2.8	34.26±2.0	43.42±2.0	35.56±1.9
pH	45 min	6.38±0.13	6.41±0.07	6.28±0.14	6.52±0.05
	24 h	5.31±0.04	5.43±0.04	5.23±0.02	5.39±0.03
L*	45 min	50.64±1.0	49.65±0.6	46.56±0.4	45.40±1.0
	24 h	56.23±2.0	54.43±0.6	54.51±0.7	54.08±0.9
a*	45 min	16.83±0.6	14.94±0.3*	14.08±0.6	15.08±0.4
	24 h	17.19±0.6	15.46±0.5*	16.48±0.5	17.12±0.5
b*	45 min	3.89±0.2	3.64±0.4	3.28±0.3	2.94±0.1
	24 h	7.02±0.4	6.33±0.3	8.04±0.6	8.21±1.0

Within stages, means with * differ (*P* < 0.05) and those with ** differ (*P* < 0.01)

Within the same line, # means the effects of dbcAMP in experiment 1 is differ from experiment 2

### Effect of db-cAMP on meat quality of finishing pigs

There were no differences on meat quality traits (e.g., pH value, drip loss, shear force, and meat color) between the control and db-cAMP-supplemented pigs in either experiment except for the decreased a* value of the meat in experiment 1 (Table 2).

### Effect of db-cAMP on biochemical indices in serum

Dietary supplementation with db-cAMP did not affect the plasma concentrations of cholesterol, TG, FFA, HDL, or LDL and the activity of lipase in either experiment. Dietary supplementation with db-cAMP increased the plasma concentrations of GH, IGF-I, T3, T4, and cAMP in experiment 1 and strikingly decreased the plasma concentration of leptin in experiment 2 (Table 3).

### Effect of db-cAMP on adipose tissue histology

**Fig. 1:**
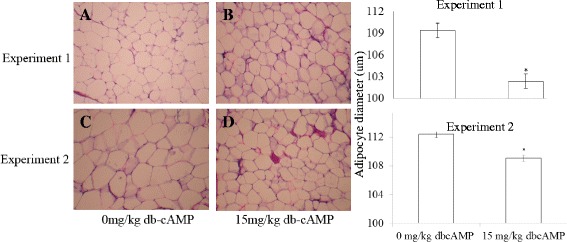
*Left*: Histological analysis of backfat in pigs at the end of experiment 1 (**a** 0 mg/kg db-cAMP; **b** treated with 15 mg/kg db-cAMP) and at the end of experiment 2 (**c** controls; **d** db-cAMP). Means are for three replicates. *Right*: The adipocyte diameter of the finishing pigs in both experiments. The control is 0 mg/kg db-cAMP, and the treatment group is 15 mg/kg db-cAMP. ―*P < 0.05, **P < 0.01 differs significantly from control

Figure 1 showed that dietary supplementation with 15 mg/kg db-cAMP increased the number of adipocytes per field and decreased the average diameter (*P* < 0.05) of backfat cell by 4.0 % in experiment 1 and by 7.0 % in experiment 2.

**Table 3:** Effects of db-cAMP on serum biochemical indices and hormones in finishing pigs

Variable	Experiment 1		Experiment 2	
	controls	db cAMP	controls	db cAMP
Cholesterol (mmol/L)	2.45±0.08	2.34±0.11	1.96±0.11	2.08±0.06
TG (mmol/L)	0.36±0.04	0.38±0.04	0.28±0.02	0.29±0.03
Lipase (U/L)	48.3±6.6	62.1±13.5	42.7±3.6	69.9±15.2
FFA (□mol/L)	142.7±18.2	253.8±25.2	203.1±34.9	316.1±90.1
HDL (mmol/L)	0.54±0.08	0.58±0.06	0.71±0.05	0.77±0.03
LDL (mmol/L)	1.52±0.09	1.48±0.06	1.30±0.08	1.36±0.06
GH (ng/mL)	8.79±0.34	11.92±0.67*	8.87±0.24	9.26±0.65
IGF-1 (ng/mL)	337±23.5	431±24.2*	333±46.5	337±44.8
IGFBP3 (ng/mL)	63.8±5.7	86.6±10.0	67.6±12.4	68.4±13.2
T3 (ng/mL)	8.46±1.19	16.27±4.15*	11.35±1.17	11.37±1.65
T4 (ng/mL)	2.81±0.09	4.13±0.52*	3.41±0.19	3.42±0.35
Leptin (ng/mL)	1.44±0.44	0.99±0.23	7.15±1.45	2.10±0.22*
AD (ng/mL)	17.31±1.59	23.53±3.48	12.65±1.33	13.83±2.09
Insulin (ng/mL)	1.98±0.03	1.81±0.05	2.01±0.03	2.01±0.04
cAMP (pmol/mL)	73.69±3.69	127.36±28.8*	85.41±9.49	87.98±3.22

Within stages, means with * differ (*P* < 0.05)

TG, triglyceride; FFA, Free fatty acid; HDL, High-density lipoprotein; LDL, Low-density lipoprotein; IGFBP3, Insulin-like growth factor binding protein 3; T3, Triiodothyronine; T4, Thyroxine; AD, Adrenaline

### Effect of db-cAMP on gene expression in hypothalamus, pituitary gland, and liver of finishing pigs

Changes in relative transcript abundance of genes of particular interest in the hypothalamus, pituitary, and liver are presented in Table 4. Treatments with db-cAMP increased the relative abundance of *ADCYAP1*, *GHRH*, *CRH*, *POMC*, and *PC1* (all *P* < 0.05) in the hypothalamus in experiment 1. Similar increases also occurred in pituitary abundance of *GHRHR* (*P* < 0.05), *GH* (*P* < 0.01), and *POMC* (*P* < 0.05) and hepatic expression of *GHR* and *IGF-1* (*P* < 0.01) in experiment 1. At the completion of experiment 2, db-cAMP treatment increased the relative abundance of *GHRH* (*P* < 0.05), *CRH* (*P* < 0.05), *POMC* (*P* < 0.01), and *PC1* (*P* < 0.05) in hypothalamus, *GH* (*P* < 0.05) and *POMC* (*P* < 0.05) in the pituitary gland, and hepatic expression of *IGF-1* (*P* < 0.05). The effect of db-cAMP on these gene expression was greater in experiment 1 than those in experiment 2 (*P* < 0.05), and db-cAMP increased the gene expressions of *GHRH*, *CRH*, *PC1*, *PC2*, *GHRHR*, *GH*, *POMC*, *GHR* and IGF-I by 173, 63, 142, 57, 32, 140, 131, 93, and 128 % in experiment 1 and 89, 45, 55, 15, 0, 115,95, 8, and 93 % in experiment 2.

**Table 4:** Effects of db-cAMP on relative gene expression in the hypothalamus, pituitary gland and liver of finishing pigs

Variable	Experiment 1		Experiment 2	
	controls	db cAMP	controls	db cAMP
Hypothalamus				
*ADCYAP1*	0.56±0.02	0.78±0.02*	0.41±0.02	0.59±0.02
*GHRH#*	0.51±0.04	1.39±0.05**	0.38±0.05	0.72±0.08*
*CRH#*	0.94±0.03	1.53±0.06*	0.66±0.02	0.97±0.05*
*POMC*	0.81±0.01	1.00±0.01*	0.46±0.03	1.81±0.01**
*PC1#*	0.33±0.02	0.80±0.05*	0.64±0.04	0.99±0.01*
*PC2#*	0.35±0.02	0.55±0.03	0.71±0.02	0.82±0.01
Pituitary gland				
*GHRHR#*	0.72±0.01	0.95±0.01*	0.45±0.01	0.46±0.01
*GH#*	0.50±0.02	1.20±0.01**	0.39±0.01	0.84±0.03*
*CRHR*	0.59±0.03	0.62±0.03	0.59±0.01	0.75±0.08
*POMC#*	0.49±0.02	1.13±0.01*	0.42±0.01	0.82±0.01*
*TRHR*	0.51±0.01	0.58±0.03	0.39±0.02	0.53±0.01
*TSH*	1.44±0.04	1.49±0.02	0.66±0.04	0.88±0.03
Liver				
*GHR#*	0.55±0.01	1.06±0.03**	0.86±0.01	0.93±0.05
*IGF-1#*	0.50±0.01	1.14±0.01**	0.57±0.01	1.10±0.02*

Within stages, means with * differ (*P* < 0.05) and those with ** differ (*P* < 0.01).

Within the same line, # means the effects of dbcAMP in experiment 1 is differ from experiment 2.

### Microarray analysis of backfat

To better understand the mechanism of the effect of dietary db-cAMP on fat deposition of the pigs, the backfat samples of experiment 1 were examined by microarrays and SAS analysis with thresholds for low probability values (FDR) set at *P* < 0.05 and log-fold change >1. Principal component analysis showed that differences between the control and treated pigs had high similarity, especially within the three control replicates.

**Fig. 2:**
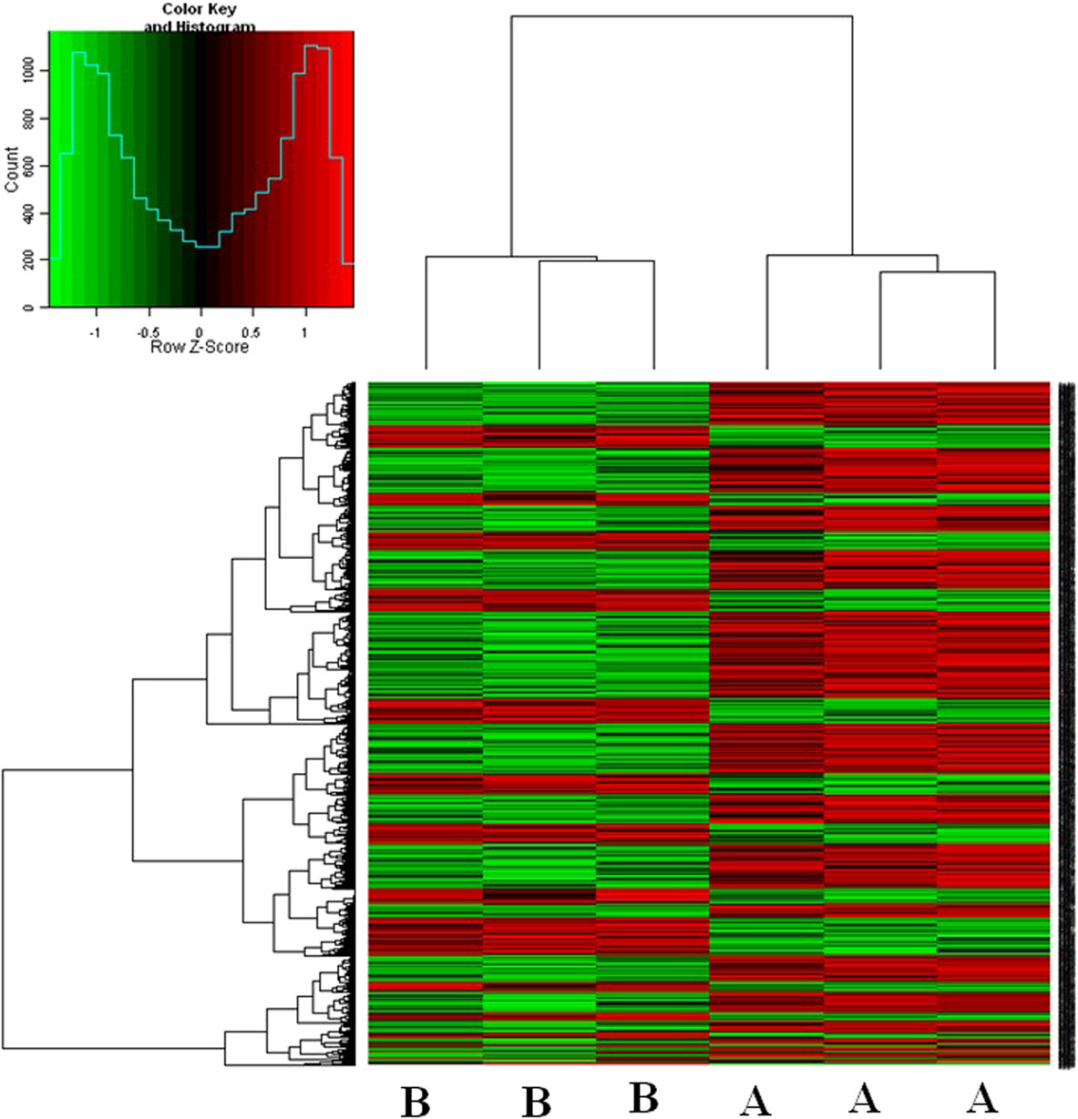
Unsupervised hierarchical clustering analysis heatmap. **a** Control samples. **b** db-cAMP samples. Three replicates (each being pools from three pigs) were analyzed for each group. *Green*: genes are down-regulated; *red*: genes are up-regulated

Compared with the controls, the expression of 739 gene sets in treated animals changed significantly (*P* < 0.05, fold change >2, or <0.5); 248 gene sets were up-regulated (red), and 491 gene sets were down-regulated (green). Of the 739, only 84 gene sets have detailed comments and can be found in public databases for pigs; 14 were up-regulated and 70 down-regulated (Table 5). The heatmap plot (Fig. 2) showed that most of these genes (83 %) were down-regulated in pigs supplemented with db-cAMP compared with the controls.

**Table 5:** The differentially expressed genes in adipocytes of finishing pigs treated with db-cAMP

Gene ID	Gene name	Gene description	P value	Fold change
Up-regulated				
396739	*SAL1*	salivary lipocalin	0.0025	22.14
407247	*LHCGR*	luteinizing hormone/choriogonadotropin receptor	0.044	5.18
641359	*SULT2A1*	sulfotransferase family, cytosolic, 2A, dehydro- epiandrosterone (DHEA)-preferring, member 1	0.0412	4.37
396597	*STAR*	steroidogenic acute regulatory protein	0.0043	4.14
403149	*CYP2A19*	cytochrome P450 2A19	0.0096	3.40
397290	*HOXD10A*	homeobox protein A10	0.0209	2.87
100124377	*SBAB-707F1.10*	solute carrier family 44, member 4	0.0353	2.58
397670	*RH*	Rh protein	0.0444	2.56
733668	*DIRAS3*	DIRAS family, GTP-binding RAS-like 3	0.0036	2.41
397324	*ACACA*	acetyl-Coenzyme A carboxylase alpha	0.0252	2.40
396861	*LCN1*	lipocalin 1 (tear prealbumin)	0.0062	2.29
100048933	*ZFAND5*	zinc finger, AN1-type domain 5	0.0113	2.25
100216478	*TEF1*	transcriptional enhancer factor 1	0.0200	2.19
414420	*RPS28*	ribosomal protein S28	0.0228	2.10
Down-regulated				
100157574	*LOC100157574*	similar to family with sequence similarity 49, member B	0.0022	0.50
100156398	*LOC100156398*	mini-chromosome maintenance complex component 4	0.0333	0.50
100154512	*LOC100154512*	similar to tripartite motif-containing 38	0.046	0.49
397330	*XPO1*	exportin 1 (CRM1 homolog, yeast)	0.0159	0.49
396599	*MCSF ALPHA*	macrophage colony stimulating factor alpha	0.0219	0.49
733579	*LOC733579*	tripartite motif protein TRIM5	0.0241	0.49
396655	*STAT1*	signal transducer and activator of transcription 1	0.0379	0.49
397177	*DUOX1*	dual oxidase 1	0.0234	0.48
100157208	*LOC100157208*	similar to Tectonic-3	0.0314	0.48
397222	*MEOX2*	mesenchyme homeobox 2	0.0095	0.48
100125540	*SYK*	spleen tyrosine kinase	0.0183	0.48
397108	*GP91-PHOX*	NADPH oxidase heavy chain subunit	0.0339	0.48
100154251	*LOC100154251*	similar to pleckstrin 2	0.0409	0.47
100048944	*CPEB1*	cytoplasmic polyadenylation element binding protein 1	0.0375	0.47
100158101	*LOC100158101*	similar to Rho GTPase-activating protein 30	0.0053	0.47
100048955	*C5AR1*	complement component 5a receptor 1	0.0121	0.47
100037274	*TAF4B*	TAF4b RNA polymerase II, TATA box binding protein (TBP)-associated factor, 105 kDa	0.0089	0.46
397041	*LGALS4*	lectin, galactoside-binding, soluble, 4	0.0439	0.46
100049698	*SNCAIP*	synuclein, alpha interacting protein	0.0078	0.45
595128	*OAS2*	2'-5'-oligoadenylate synthetase 2, 69/71 kDa	0.0401	0.453
397422	*CCL2*	chemokine (C-C motif) ligand 2	0.0181	0.45
387287	*HCRTR1*	hypocretin (orexin) receptor 1	0.0387	0.45
100124526	*CD1D*	CD1d molecule	0.0189	0.45
397271	*AIF1*	allograft inflammatory factor 1	0.0132	0.44
396935	*PCCB*	propionyl Coenzyme A carboxylase, beta polypeptide	0.0326	0.44
733649	*TAP1*	transporter 1, ATP-binding cassette, sub-family B (MDR/TAP)	0.0419	0.44
100156191	*LOC100156191*	zygote arrest 1-like protein	0.0402	0.44
414912	*PU.1*	transcription factor PU.1	0.0044	0.43
100157355	*LOC100157355*	similar to spindle and KT associated 1	0.0325	0.43
404704	*CD4*	CD4 molecule	0.0029	0.43
100126851	*SERPINB9*	serpin peptidase inhibitor, clade B (ovalbumin), member 9	0.0464	0.42
396723	*DDX58*	DEAD (Asp-Glu-Ala-Asp) box polypeptide 58	0.0049	0.42
396943	*ITGB2*	integrin, beta 2 (complement component 3 receptor 3 and 4 subunit)	0.0088	0.41
397141	*NPL*	N-acetylneuraminate pyruvate lyase	0.0157	0.40
100037296	*TLR7*	toll-like receptor 7	0.0053	0.40
100048946	*RNASEL*	Ribonuclease-L(2',5'-oligoisoadenylate-synthetase- dependent)	0.0105	0.40
100048958	*GNA14*	guanine nucleotide binding protein (G protein), alpha 14	0.0486	0.40
397441	*CD86*	CD86 molecule	0.0208	0.40
399500	*IL6*	interleukin 6 (interferon, beta 2)	0.0176	0.39
397590	*PGHS-2*	prostaglandin G/H synthase-2	0.024	0.38
100049665	*KRT2*	keratin 2	0.019	0.38
445460	*C1QC*	complement component 1, q subcomponent, C chain	0.0045	0.38
100156254	*LOC100156254*	similar to Plastin-2 (L-plastin) (Lymphocyte cytosolic protein 1) (LCP-1) (LC64P)	0.0435	0.38
100155269	*LOC100155269*	similar to lymphocyte antigen 9	0.0131	0.37
396900	*AMCF-II*	alveolar macrophage-derived chemotactic factor-II	0.0494	0.37
404772	*GEM*	GTP binding protein overexpressed in skeletal muscle	0.0291	0.36
397414	*UF*	uteroferrin	0.0066	0.36
100153090	*LOC100153090*	similar to Cathepsin S	0.0411	0.36
414373	*CCR3*	chemokine (C-C motif) receptor 3	0.0179	0.3
100152784	*LOC100152784*	Vascular non-inflammatory molecule 3 precursor (Vanin-3)	0.0392	0.35
399541	*TLR4*	toll-like receptor 4	0.0474	0.35
606745	*LOC606745*	A2b adenosine receptor	0.0159	0.35
100049673	*HPS3*	Hermansky-Pudlak syndrome 3	0.038	0.35
396624	*OX40L*	OX40L protein	0.0323	0.35
414374	*CCR1*	chemokine (C-C motif) receptor 1	0.0196	0.34
100152550	*LOC100152550*	similar to EF-hand domain (C-terminal) containing 1	0.038	0.34
503658	*ENPP2*	ectonucleotide pyrophosphatase/phosphodiesterase 2	0.0481	0.33
397079	*CECR1*	cat eye syndrome chromosome region, candidate 1	0.0057	0.33
396880	*IL8*	interleukin 8	0.0091	0.32
396985	*PLAU*	plasminogen activator	0.0474	0.32
100154994	*LOC100154994*	similar to serine proteinase inhibitor, clade B, member 10	0.0192	0.31
574057	*PDCD1LG2*	programmed cell death 1 ligand 2	0.0178	0.30
396726	*FASLG*	Fas ligand (TNF superfamily, member 6)	0.0261	0.30
414904	*CXCL2*	chemokine (C-X-C motif) ligand 2	0.0093	0.30
100156373	*LOC100156373*	similar to villin 1	0.0067	0.28
397373	*MYO7A*	myosin VIIA	0.0473	0.27
397102	*AMBN*	ameloblastin	0.0329	0.19
100049669	*GPNMB*	glycoprotein (transmembrane) nmb	0.0447	0.18
396662	*CD2*	CD2 molecule	0.0212	0.16
100135044	*SBAB-591C4.5*	MHC class II, DQ alpha	0.0027	0.09

**Fig. 3:**
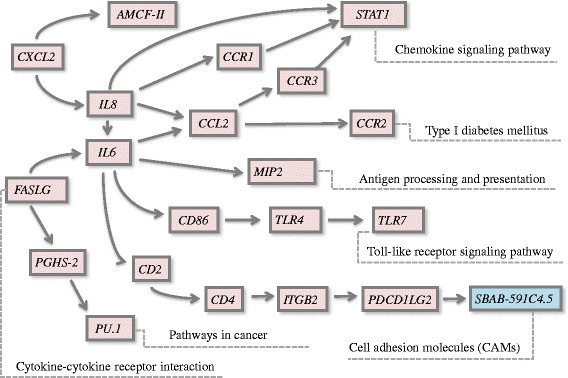
Pathway analysis of the differently expressed genes involved in several changed pathways of the db-cAMP treatment group compared with the control group. *Green*: genes are down-regulated; *red*: genes are up-regulated

Gene ontology analysis (SAS) showed that, compared with the controls, differentially expressed genes in db-cAMP supplemented pigs were significantly enriched in the categories listed in Table 6. The highest enrichment occurred in cell activation and immune functions, including leukocyte functions. Pathway analysis of the 84 differentially expressed genes in pigs supplemented with db-cAMP exposed the top five significant pathway maps involving 30 genes, and all of these were down-regulated (Fig. 3). The pathways, chemokine signaling, cell adhesion molecules (CAMs), Toll-like receptor signaling, cytokine-cytokine receptor interaction, antigen processing and presentation, and type I diabetes mellitus are consistent with the dietary supplementation causing a significant change in immune or inflammatory status of the adipose tissue.

**Table 6:** Significant GO of differentially expressed genes

GO ID	Annotation	Number Changed/number measured (%)	Enrichment test p-value	Q value
GO:0001775	cell activation	15/150 (10)	0.0001	0.0202
GO:0050900	leukocyte migration	7/42 (16.67)	0.0005	0.0501
GO:0045321	leukocyte activation	12/123 (9.76)	0.0005	0.0501
GO:0042330	Taxis	9/76 (11.84)	0.0007	0.0569
GO:0002376	immune system process	24/410 (5.85)	0.0010	0.0681
GO:0001816	cytokine production	8/90 (8.89)	0.0072	0.3915
GO:0048583	regulation of response to stimulus	13/206 (6.31)	0.0101	0.4143
GO:0045058	T cell selection	3/16 (18.75)	0.0168	0.4460
GO:0009605	response to external stimulus	22/456 (4.82)	0.0168	0.4460
GO:0002682	regulation of immune system process	11/173 (6.36)	0.0170	0.4460
GO:0030029	actin filament-based process	10/154 (6.49)	0.0200	0.4460
GO:0006955	immune response	15/277 (5.42)	0.0205	0.4460
GO:0002520	immune system development	10/161 (6.21)	0.0260	0.5319
GO:0043900	regulation of multi-organism process	2/10 (20)	0.0463	0.7559

### Verification test

**Fig. 4:**
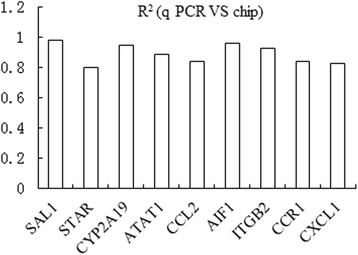
The correlation coefficient of selected genes expressing level by RT-qPCR and gene chip data. *Vertical axis*: the value of correlation coefficient of the gene expression level by RT-qPCR and gene chip data; *horizontal axis*: detected genes

A selection of ten genes with relatively large change and related to fat metabolism or cell signaling were chosen to verify that their relative transcript abundance determined by the gene chips could be confirmed using real-time, quantitative PCR. For nine genes (Fig. 4), there was high correlation between the results of the mRNA array and qPCR methods (the average *R*^2^ 89 %).

## Discussion

Consistent with others’ findings [39], the present dietary supplementation with 15 mg/kg db-cAMP significantly decreased the backfat thickness and fat percentage of finishing pigs in both experiments while it did not affect growth performance or meat quality in either experiment, but Tian [36] found that db-cAMP stimulated the growth performance of pigs. The present research also showed that db-cAMP decreased the backfat thickness and backfat percentage more marked in the earlier stages than the later stages of finishing pigs, which implied that supplementation of db-cAMP in the earlier phase is better than the later phase of finishing pigs. This additive also was found to improve carcass composition and increase longissimus area and lean percentages in other experiment [39]. In our experiment, the longissimus area had a trend to increase after treated with db-cAMP. The changes in adipocyte volume can be used to estimate fat deposition in pigs [33] and adult rats [24]. The result of our experiment verified the previous found that dietary db-cAMP decreased the diameter of adipocytes [38] in two different stages of finishing pigs, which may be caused by inhibiting the proliferation of preadipocytes and their differentiation [21], but the mechanism of db-cAMP inhibiting the proliferation and differentiation of preadipocytes is not illuminated yet. Db-cAMP increasing the activities of lipolytic enzymes [4] and inhibiting lipogenesis [21] was another reason for the backfat thickness decreased. The present study found that db-cAMP strikingly increased the plasma concentrations of cAMP at the earlier phase of finishing pigs and decreased the plasma concentrations of leptin at the later phase of finishing pigs, which verified the finding of Maeda and Horiuchi [19].

Dietary db-cAMP clearly influenced fat metabolism related to hormone and genes in the hypothalamus and pituitary, most notably in the earlier phase of finishing pigs; this is our novel founding. Both GH and its downstream peptide IGF-I and T3 and T4 were at higher concentrations in db-cAMP-treated pigs. Underlying these changes, there were increases in several gene expression level in the hypothalamus, pituitary, and liver in supplemented pigs. These included hypothalamic expression of *ADCYAP1* which activates adenylyl cyclase to increase endogenous cAMP and stimulates secretion of *GHRH* and *CRH* [3, 20], *GHRH*, *CRH*, *POMC*, and *PC1* genes. Supplementation with db-cAMP increased pituitary expression of *GHRHR* and *GH* and hepatic expression of *GHR* and *IGF-1*, all of which indicated enhanced activity of the GHRH-GH-IGF-1 axis. Some of these responses were also evident, though of less magnitude, in the second stage of finishing (day 170), consistent with higher circulating concentrations of GH and IGF-1 in the supplemented pigs. In experiment 1, supplemented pigs had higher plasma concentrations of T3 and T4 though no changes in pituitary expression of *TRHR* or *TSH*. The thyroid hormones promote and, in cooperation with GH and IGF-1, stimulate lipolysis [8, 37]. The findings described here help explain that db-cAMP increased the plasma contents of GH, IGF-1, T3, and T4 [6, 21, 39]. Dietary supplementation with db-cAMP also influenced the POMC axis with increasing the expression of *POMC* both in hypothalamus and pituitary and *CRH*, *PC1*, and *PC2* in hypothalamus. This system is implicated in the regulation of lipolysis and interactions between CRH and POMC [2, 9, 28], which also was affected by the dietary energy level and energy intake [22] and hydrolyzed by PC1 and PC2 [14, 34]. The changes detected here suggest some involvement of the POMC system in the effects of db-cAMP on lipid metabolism and fat accretion, and the possible mechanisms need to be further researched.

Db-cAMP decreasing the backfat thickness and backfat percentage was verified by the 2 experiments using different phases of finishing pigs in this present study, and we found new mechanism of db-cAMP regulating fat metabolism by mRNA array analysis that was db-cAMP affected the immune/inflammatory reaction. As the backfat thickness was obviously reduced by dietary db-cAMP in the earlier phase of the finishing pigs, gene expression in this tissue also was examined to expose likely underlying mechanisms. Go analysis result showed that db-cAMP inhibited the proliferation and differentiation of fat cells and reduces the deposition of adipose tissue through inhibiting adipose tissue cell activation, cell tropism, leukocyte activation, migration, and the immune system activation of the fattening pig. A relatively low proportion (11 %) of the differentially expressed gene sets was adequately documented, and of these, the bulk (83 %) was down-regulated in db-cAMP-treated pigs. It was of interest that the most striking changes occurred, not in genes obviously related to lipid metabolism but in cohorts associated with immune/inflammatory functions related to insulin sensitive or lipolysis. There is increasing recognition that inflammatory reactions in adipose tissue contribute to adiposity and appear to be linked to the metabolic syndrome and type I diabetes. The differentially expressed genes identified here were most obviously associated with pathways including chemokine signaling, CAMs, antigen processing and presentation, Toll-like receptor signaling, cancer, cytokine-cytokine receptor interaction, and type I diabetes mellitus. The chemokine member *CCL2* was highly expressed in adipose tissue and contributed to the cells becoming insulin resistant [10, 26] while *CXCL2* (or *MIP-2*), encoding macrophage inflammatory protein 2-alpha (MIP2-alpha), was the main marker of inflammatory reaction in metabolic syndrome [16, 35]. Another cytokine IL-8 [18] was highly expressed in hypertrophied adipocytes [1], and its inhibition decreased the likelihood of obesity and type 2 diabetes [15]. Cytokine receptors such as *CCR1*, *CCR2*, *CCR3*, and *CCR5* also related to insulin sensitivity [12], in turn closely connected with the size of adipocytes [32]. These genes also were down-regulated and stimulated lipolysis [23]. Although in the present experiment db-cAMP did not decrease the concentration of plasma insulin, it could decrease the insulin sensitivity and stimulate the lipolysis, which reasons need further research. The CAMs pathway included a number of differentially expressed genes involved in inflammatory infiltration, reactions, and responses [25]. Their down-regulation in pigs treated with db-cAMP suggests diminished inflammatory sensitivity, perhaps resulting in reduced fat accretion. This interpretation was further supported by the diminished expression of several genes involved in Toll-like receptor signaling pathways critically involved in mediating inflammatory responses including in adipocytes, some of which were readily influenced by high-energy diets [27] and contributed to other pro-inflammatory factors secretion and insulin resistance [13]. The differential expression of these genes in backfat exposed by microarray analysis was, for the most part, convincingly supported by quantitative PCR measurements so they appear to be legitimate.

## Conclusions

The results obtained from these analyses indicated that dietary supplementation of finishing pigs with db-cAMP resulted in significantly reduced accumulation of subcutaneous fat without changing growth performance or carcass characteristics, apparently stemmed from suppression of the inflammatory system within the adipose tissue related to insulin sensitive or lipolysis. There were additional systemic effects of treatment detected within major endocrine GHRH-GH-IGF-1 axis and POMC system, which are known to be involved in growth and energy metabolism. Any reduction in excessive fat deposition in finishing animals has economic and food quality consequences, so improved understanding of the underlying mechanisms allows exploring new strategies for manipulating fat accretion. This study has contributed to explaining how dietary supplemental db-cAMP provides a potential approach to achieving a more desirable animal product. In addition, this study illustrated that db-cAMP regulated fat deposition through improving the immune system, which is of potential value to dissect molecular pathways influencing fat deposition by db-cAMP. It would be benefit for health of human clinically used for weight loss.

## Abbreviations

AD: adrenaline
*ADCYAP1*: adenylate cyclase activating polypeptide
*CRH*: corticotropin releasing hormone
*CRHR*: corticotropin releasing hormone receptor
db-cAMP: dibutyryl-cAMP
*GH*: growth hormone
*GHR*: growth hormone receptor
*GHRH*: growth hormone releasing hormone
*GHRHR*: growth hormone releasing hormone receptor
*IGF-1*: insulin-like growth factor-1
IGFBP3: insulin-like growth factor binding protein 3
*PC*: prohormone convertase
*POMC*: pro-opiomelanocortin
T3: triiodothyronine
T4: thyroxine
*TRHR*: thyrotropin-releasing hormone receptor
*TSH*: thyroid stimulating hormone
